# Increasing understanding of the relationship between geographic access and gendered decision-making power for treatment-seeking for febrile children in the Chikwawa district of Malawi

**DOI:** 10.1186/s12936-016-1559-0

**Published:** 2016-10-24

**Authors:** Victoria L. Ewing, Rachel Tolhurst, Andrew Kapinda, Esther Richards, Dianne J. Terlouw, David G. Lalloo

**Affiliations:** 1 Malawi-Liverpool-Wellcome Trust Clinical Research Programme, Queen Elizabeth Central Hospital College of Medicine, P.O. Box 30096, Blantyre, 3 Malawi; 2 Liverpool School of Tropical Medicine, Pembroke Pl, Liverpool, L3 5QA UK

**Keywords:** Malaria, Treatment-seeking, Gender, Intra-household decision-making, Malawi

## Abstract

**Background:**

This study used qualitative methods to investigate the relationship between geographic access and gendered intra-household hierarchies and how these influence treatment-seeking decision-making for childhood fever within the Chikwawa district of Malawi. Previous cross-sectional survey findings in the district indicated that distance from facility and associated costs are important determinants of health facility attendance in the district. This paper uses qualitative data to add depth of understanding to these findings by exploring the relationship between distance from services, anticipated costs and cultural norms of intra-household decision-making, and to identify potential intervention opportunities to reduce challenges experienced by those in remote locations. Qualitative data collection included 12 focus group discussions and 22 critical incident interviews conducted in the local language, with primary caregivers of children who had recently experienced a febrile episode.

**Results:**

Low geographic accessibility to facilities inhibited care-seeking, sometimes by extending the ‘assessment period’ for a child’s illness episode, and led to delays in seeking formal treatment, particularly when the illness occurred at night. Although carers attempted to avoid incurring costs, cash was often needed for transport and food. Whilst in all communities fathers were normatively responsible for treatment costs, mothers generally had greater access to and control over resources and autonomy in decision-making in the matrilineal and matrilocal communities in the central part of the district, which were also closer to formal facilities.

**Conclusions:**

This study illustrates the complex interplay between geographic access and gender dynamics in shaping decisions on whether and when formal treatment is sought for febrile children in Chikwawa District. Geographic marginality and cultural norms intersect in remote areas both to increase the logistical and anticipated financial barriers to utilising services and to reduce caretakers’ autonomy to act quickly once they recognize the need for formal care. Health education campaigns should be based within communities, engaging all involved in treatment-seeking decision-making, including men and grandmothers, and should aim to promote the ability of junior women to influence the treatment-seeking process. Both mothers’ financial autonomy and fathers financial contributions are important to enable timely access to effective healthcare for children with malaria.

## Background

Malaria remains a major contributor to childhood morbidity and mortality in sub-Saharan Africa. Artemisinin-based combination therapy (ACT) is highly efficacious, and prompt case management is essential to reduce the global burden of malaria. This relies on identifying and removing barriers to accessing treatment, and developing interventions to increase uptake of services. There is a growing body of literature to support the finding that household structure and power relations, and in particular restrictions on women’s decision-making authority, has a considerable impact on treatment-seeking for malaria [1–4] and on poor child health outcomes in general [5–7]. A recent systematic review of qualitative research on household recognition and response to child diarrhoea, pneumonia and malaria found that both intra-household relations and structural barriers to formal healthcare were common influences on decisions to seek treatment beyond the household and community in sub-Saharan Africa [8]. However, the review highlighted a lack of research exploring how context, including geographic location, shapes the impact of these influences. There are also increasing calls for a deeper understanding of the intersections between gender and other axes of disadvantage and marginality such as geographic location and poverty [9]. To date, few studies have examined how gendered intra-household dynamics interact with other axes of disadvantage and marginality in relation to health to influence treatment-seeking behaviour for children. This paper aims to contribute towards understanding in this area by exploring how mothers’ autonomy, access to and control over resources interact with their geographic access to services to shape the outcome and timing of decision-making processes regarding care-seeking beyond the village.

This study was set within the culturally heterogeneous Chikwawa district of Malawi: a region home to a number of ethnic groups. Quantitative research in the Chikwawa district found that compared to those living near the hospital, those living in remote villages were less likely to attend a health facility, more likely to delay attendance at health facility and experienced greater costs associated with attending a health facility [10]. The findings correlate with studies conducted elsewhere in identifying distance from facility and costs of utilization as important determinants of service utilization for childhood febrile illness [4, 10–12]. One notable feature of the district is differences in traditions regarding descent (patrilineal versus matrilineal) and marital residence (matrilocal versus patrilocal) between different ethnic groups. These traditional differences interrelated with distance because those nearer the centre of the district tended to be matrilocal and matrilineal, whilst those in remote areas tended to be patrilineal and patrilocal. Such differences might influence decision-making processes and the relative autonomy of carer-givers. Qualitative methods were therefore used to add depth of understanding to the previous quantitative findings. Earlier qualitative work in the district examined how decision-making on whether and when to seek care outside the home in this setting is guided by interpretation of illness causation and severity [13]. This paper presents qualitative findings that explore how gender and generational dynamics in the decision-making process interact with geographic access to services and anticipated costs to further add to the complexity of the treatment-seeking decision-making process and delays in seeking care. Potential intervention opportunities are discussed, that target these inter-connected challenges, particularly experienced by those in remote locations.

## Methods

### Study setting

This paper and the previous quantitative study were both part of a larger a community-based trial investigating the programmatic implementation of ACT in children below 5 years of age across 50 villages within the Chikwawa district (Clinical Trial ID No: NCT010380632). This district suffers from a high burden of malaria cases; malaria is endemic with perennial transmission. The Shire River, the main outlet of Lake Malawi, travels through the district making large areas prone to flooding. Public health care in the district is provided by the district hospital, one rural hospital, one mission hospital, 14 health centres and 26 community health worker (CHW) operated health posts, which cater for the district’s population of approximately 450,000. There are also a number of private clinics. Clinic visits, diagnosis and treatment are free at the point of delivery through the public health system, although stock-outs occur. At the time of the study ACT was not available through CHW-operated health posts in the Chikwawa district, which operated a referral system for children suffering from febrile illness.

The Chikwawa district is characterized by the presence of a number of ethnic groups. Around the central area of the district the Chewa and Mang’anja ethnic groups predominate. This is a trading area, so the people are exposed to external influences and ideas. The Sena ethic group are the majority in remote areas of the district. Chewas and Mang’anjas traditionally form a matrilineal and matrilocal society, whereby the husband leaves his home area to live with his wife’s family; whereas the Sena are patrilineal and patrilocal [14–16]. Although males take the role of family head in both family structures, in matrilocal and matrilineal communities women are surrounded by their natal extended families rather than those of their husbands and, therefore, may be able to draw on their support.

### Procedures

Qualitative data collection was conducted between September 2010 and February 2011. Four local fieldworkers conducted the qualitative data collection in the local language (*Chichewa*, although some *Chisena* was used in remote villages). 12 focus group discussions (FGDs) with male and female community members and 22 critical incident interviews (CIIs) were carried out with primary caregivers of children who had recently experienced a febrile episode (Table 1). The fieldworkers undertook a week long training in qualitative research methods, with a specific focus on conducting CIIs and FGDs. Training sessions also explored issues around treatment-seeking for childhood fever. Fieldworkers conducted FGDs and CIIs with individuals of the same sex. Two rounds of FGDs were conducted. Six FGDs initially gathered information about perceived causes of fever, norms around appropriate responses to each cause and challenges experienced during treatment-seeking. Participants were given short vignettes to introduce commonly occurring scenarios around childhood fever and were asked to role-play and discuss what might happen next. This was intended to promote participants’ engagement with each other and the research topic [17, 18]. Repeat FGDs were conducted with each group to discuss earlier findings, for clarification and to add depth of understanding.

**Table 1:** Qualitative methods

Focus group discussions^a^	Area of residence		Total
	Central	Remote	
Young women^b^	2	2	4
Older women	2	2	4
Men	2	2	4
Total focus group discussions	6	6	12

Critical incidence interviews	Area of residence		Total
	Central	Remote	
Women whose child experienced fever and attended a formal health facility	7	5	12
Women whose child experienced fever and did not attend a formal health facility	3	7	10
Total critical incidence interviews	10	12	22

^a^8 to 10 participants per group

^b^Younger women’s groups were conducted with women aged between 18 and 24. These were restricted to married women with fewer than 4 children, as CAGs advised that unmarried young women may have not felt able to participate freely in groups of married women, and those with more children would assume a more senior role in the community

Maximum variation purposive sampling was used to select FGD participants, ensuring that a range of perspectives was included [19, 20]. FGD participants were selected by community advisory groups (CAGs) set up as part of the parent study. FGDs were conducted in two village clusters, representing two diverse sub-sets of the population in the Chikwawa district - those living around the commercial centre of the district, and those living in remote areas. The remote areas selected for FGDs were villages defined by the Ministry of Health as being ‘hard-to-reach’ on the basis of being more than 8 km from a health facility or due to the presence of geographical obstacles. Discussions held with CAGs during selection emphasized the importance of identifying ‘symbolically’ representative members of the community; criteria included selecting individuals with metal and straw roofs; those who lived closer and farther from the village centre; and those from different family groups. The team was aware of the likelihood that friends and relatives might be selected, however, previously collected household wealth data suggested that within each sub-community, individuals were fairly homogenous in terms of possession ownership as a proxy for wealth [10]. Separate groups were conducted with younger women, older women and men of mixed ages. Younger women’s groups were restricted to married women approximately between the age of 18 and 24 with fewer than four children. Ages were approximate because people in Malawi do not always remember the exact year of their birth. CAGs advised that unmarried young women may have not felt able to participate freely in groups of married women, and those with more children would assume a more senior role in the community.

CIIs took the form of narratives through which individuals gave their account of their child’s illness from recognition of illness to time of recovery or interview. A continuous (‘rolling’) multiple indicator survey (MIS) with monthly data collection was conducted as part of the parent study [21]. A malaria rapid diagnostic test (RDT) was conducted for all surveyed children. CIIs were conducted with two groups of MIS participants. The first group included primary caregivers whose child was RDT positive, where fever had been recognized for more than 24 h before the time of the survey and whose child had not attended a formal health facility. The second group included primary caregivers whose children had received recent malaria treatment from a health facility, with or without a positive malaria test. The 24-h period was used to define those who had not yet sought care, since they may have gone on to do so within the recommended period if the survey team had not attended their house. Those who tested positive for malaria and who had not already received treatment were treated by the study team. Individuals in both groups were considered to have had probable malaria, enabling comparison of treatment-seeking between those who had attended a formal health facility and those who had not. It would not have been possible to sample sufficient numbers of individuals whose child had recent malaria only from the most remote and close villages within the timeframe, so CIIs were sampled across all the villages included in the parent study. CIIs were classified as central if they were conducted with individuals living in villages located within 15 min’ walking distance of the district hospital, and remote if they lived further away. CIIs were used to explore the relationship between intra-household decision-making, the accessibility of facilities and cultural practices. The principle of saturation was used; data collection continued until the research team was confident that no new information (categories and themes) was emerging and they understood the issues being expressed [19, 22].

Detailed topic guides were used to focus the CII and FGDs on the areas of interest and ensure all the relevant topics were covered [19]. However, the fieldworkers used the topic guide flexibly, asking questions in any order and adding questions to gain more detail or explore topics of interest [23]. FGD participants were asked to discuss the different perceived causes of fever and appropriate responses to each. The treatment-seeking decision-making process was then explored including asking about which individuals were involved, what each person’s role was and why. Draft topic guides were reviewed and refined with the fieldworkers before and after piloting. Two small pilot FGDs were held in villages close to the field site and with three caregivers who had attended the facility for treatment. Voice recordings of pilot and all subsequent CIIs were reviewed on return from the field; translated transcripts of the pilot FGDs were discussed in detail, highlighting specific areas for encouragement and training. Transcripts of each subsequent FGD were reviewed in detail during preparation of topic guides for repeat FGDs.

### Data analysis

The team met after each round of qualitative data collection to review transcripts and discuss emerging themes. Data analysis was based on a framework approach [24]. The CIIs and FGDs were recorded, transcribed and translated into English then imported into the qualitative analysis software QSR NVivo 9 for analysis. All transcripts were read through and summaries written. A broad index was developed during coding of the first two FGDs and CIIs (by VE in discussion with AK and RT), based on themes emerging from the data. The index had the following main categories: accessibility; illness typology; disease outcome; finance; interactions; treatment type; narrative; people; perception; recognition; roles and responsibilities; signs and symptoms; treatment-seeking pathway. The index was then applied to code all data and iteratively refined throughout coding (VE and AK under guidance from RT). Data were charted by summarizing and comparing data coded under different indexes and across different categories of participants, and these charts were used to develop descriptive analyses and explanations (VE, RT and ER).

### Ethics statement

Ethical approval was obtained from the College of Medicine Research and Ethics Committee, Malawi (P.04/09/783 and P.10/08/707) and the Liverpool School of Tropical Medicine Research Ethics Committee (09.07). Additionally permission was granted from village leaders. Sensitization campaigns were conducted as part of the parent study to inform the communities about all aspects of the trial and affiliated studies. Fully informed written consent was obtained from all participants in the local language.

## Results

CII and FGD participants from both central and remote areas explained that in the majority of instances they initially spent time assessing the illness severity. Participants differentiated between “ordinary fever”, and that requiring malaria treatment, using a ‘wait and see’ approach which usually took between 2 and 5 days (A detailed analysis of this approach and perceptions of severity has been published elsewhere [13]). Participants explained that formal health facilities were the most appropriate source of care for fever assessed as requiring malaria treatment. However, their ability to seek formal healthcare and the timing of this was shaped by interactions between the geographic accessibility of facilities, related costs, access to and control over resources to meet these costs and gendered intra-household decision-making authority.

### Geographic accessibility of formal health facilities

Once the need for formal healthcare was recognized, distance from a health facility was raised in both CIIs and FGDs as a barrier to seeking timely care. Three of the CII participants explained that they had decided not to seek care from a health facility, or delayed care seeking because of the distance to a health facility. Participants in all FGDs described the time of day that illness occurred as an important consideration for care seeking, and it influenced delayed health facility attendance in a number of CII participants. Travel to a health facility at night was considered dangerous, wherever the participant lived, as it was perceived to put the primary caregiver, who was usually female, at risk of murder or mugging, or accusation of criminal activity by police. Participants stated that extremely sick children would be taken to the hospital at night, but the caregiver would require accompanying by the husband or neighbours. This issue in combination with distance from a health facility posed particular problems for those living in remote villages. Night-travel was avoided, rather participants considered whether they could feasibly travel to the hospital, receive treatment and travel back again before night-time. If not they preferred to wait and leave early the following morning, unless they perceived the child to be severely ill:

*Not many people will rush to the hospital in the first place, mostly when we consider the distance to be covered. Supposing the child is sick now, I can’t go to Chikwawa and come back. It will be too late. Usually, we wait first because we think about the long distance. That’s our problem here.* (Men’s FGD, remote, 07/10/10).

The above quote illustrates that the distance to a facility influences the length of the assessment period in the “wait and see” approach. However, participants were aware that sometimes the illness might become serious before the need for care is recognized:

*When he is in trouble, sometimes I don’t realize that he has malaria but I just realize that he has fever… I realize that the child has collapsed; then I know that it must have started long ago but I didn’t know.* (Young women’s FGD, central, 22/01/11).

Thus the “wait and see” approach has greater potential adverse consequences for those living in remote villages due to the additional time needed to travel to the health facility once the need for care is recognized.

### Costs, access to and control over resources

Households experienced considerable financial costs associated with attending a formal health facility. Despite care that was free at the point of delivery, finances were often needed for transport and food, so money for anticipated costs was needed prior to seeking treatment. In general men were identified as responsible for budgeting and decision-making with regard to household finances in both communities. Women had limited financial autonomy, and women’s, often considerable contributions to income generation were minimized in participants’ narratives.

Some of the women explained that they had no right to *control* or contribute in decisions over finances because they were ‘just’ beneficiaries of their husbands, who were seen as the income generators. Women living in remote areas tended to express more limited control over resources, such as in the following quote:

*When he decides that the money is supposed to be used in such and such a way, I just have to obey what he says.* (CII, attended a health facility, remote, 04/11/10).

A small number of women stated that they conducted the budgeting together with their husband; it was notable that all of these women also had a source of income of their own, such as working as a vendor, and most of them were living in the central area. However, most women in all areas do have some access to finances, since they are typically responsible for selling fruits and vegetables produced by the household, beyond that needed for consumption, and for buying items necessary for daily household running.

Women and men in all areas agreed that it was a child’s father’s responsibility to provide money, if required, for treatment-seeking. In the majority of the cases discussed in CIIs husbands had fulfilled this responsibility by providing the necessary money for treatment. In addition some male and female FGD members stated that if the husband went away, he would leave money for emergencies; this was confirmed in one of the CIIs. Men may need to borrow money in order to be able to provide it for their children’s treatment. However, men did not always provide money and were sometimes unable to. Women in all areas described a variety of strategies that they used to access finances when required: this included accumulating savings by hiding some of the change when given money for purchases; selling vegetables from the garden; and borrowing from friends, family members and grocery store owners. Both men and women described giving clothing as a pledge to either traditional healers or money-lenders in exchange for treatment or cash.

Households had to balance competing demands for resources. Women initially assessed the level of need for urgent treatment and the availability of resources and only requested financial assistance, or made use of their own finances, if they deemed it necessary. In most cases out-of-pocket expenditure was avoided:

*We usually walk, not everyone will hire a bike. Most people walk because of financial constraints. Let’s imagine you only have K200 in the house and you have neither flour nor relish and the child falls ill, if you take a bicycle and go to the hospital, what will the child eat when you come back from the hospital?* (Young women’s FGD, central, 06/10/10).

The above participant continued to explain that women might take the risk of spending some of their own income on transport if the case is serious, although they ultimately expected their husband to cover the cost:

*When the sickness is very* ***serious*** *, we spend the money to go to the hospital simply because it is a* ***serious*** *case… We know that the husband will do his best to bring money home.*

Women with an independent source of income were usually in a better position to seek care without financial assistance from their husband. One CII participant living in the central area sought care for her child on the day the illness started, using a bicycle taxi funded from the proceeds of a small business she ran.

### Gender, generation and intra-household decision-making power

Women and men in all areas described a chain of communication, whereby the child’s mother usually identified childhood illness and informed the father. However, there were clear differences in the level of autonomy and authority that women possessed with regard to making treatment-seeking decisions in remote villages where patrilineal and patrilocal ethnic groups dominated versus central villages where matrilineal and matrilocal ethnic groups were in a majority. Mothers in remote villages not only had greater need for financial assistance for transport, but also experienced limited authority to make decisions about the appropriate course of action. The men in remote villages were in agreement that it is necessary for mothers to inform their husbands, or if not present, their in-laws, before seeking care for children. Paternal grandmothers were especially important in the treatment-seeking process, although if finances were required, the mother sometimes needed to wait for her husband to receive the necessary assistance. In the patrilineal/patrilocal communities in-laws were considered to ‘own’ the children and therefore the right to know what is happening and contribute to the decision-making process:

*We tell them so that they should be aware, because our customs here require that the wife should live at the husband’s home and we believe that the husband’s parents and relatives are the owners of the children*. (Young women’s FGD, remote, 14/01/11).

Within these patrilineal communities, participants were unanimous that the social consequences of mothers not informing relatives could be serious:

*Suppose the woman leaves without informing anybody because the child is ill and perhaps the child dies or on the way the child dies, in our tradition it is a big issue, she has a case to answer. The question will be: why did she have to go without informing anyone? She must have done something bad to the child* – *this may cause a row.*

Men in remote also showed great respect for elders; they suggested they would go along with the elders’ advice, even if it was contradictory to their own preferences. Unlike the women however, men were not required to seek the advice of elders before acting:

Women in remote villages explained important advantages of informing their relatives, for example informed relatives, particularly mothers-in-law, could provide any assistance necessary, such as childcare and food preparation. This was perhaps more of a concern for those living in remote villages due to the greater distance from home and greater difficulties of sending requests for help than for those living centrally. However, some women in remote villages criticized the existing system of authority and expressed frustration about delays caused by needing to inform others, with some suggesting they would be willing to disobey their seniors in order to ensure their child gets the care they need:

*I would rather be shouted at, as long as my child is well.* (Older women’s FGD, remote 081010).

Despite the challenges, women in remote villages still expressed preference for hospital treatment:

*Our generation depends much on the hospital; therefore we go to the hospital first…*(Young women’s FGD, remote, 08/10/10).

In contrast to those in remote villages, those in central villages did not refer to seeking advice from mothers-in-law. They were less likely to require financial assistance due to the closer proximity to the hospital, although they agreed that the husband has responsibility for decision-making, and should be informed of sickness. Some agreed that nearby relatives are particularly influential for women, usually her own, and explained there may be disagreements if she acted without their knowledge. However, the men in central villages considered the woman able to make decisions in the absence of the husband and in fact, that it was important for her to do so:

Respondent 2: *…it might happen that a child falls sick when the husband is at work in Nchalo and the wife decides to wait for the husband to take the child to the hospital; now can we say that the woman is wise? When a child is sick, it is the responsibility of the one who is present to take the child to the hospital.*

Respondent 1: *I can say that it is a question of responsibility.*

Respondent 4: *Yes, both have the responsibility over the child.* (Men’s FGD, central, 17/01/11).

## Discussion

This study aimed to explore how geographic location and gendered intra-household decision-making power interacted to influence treatment-seeking decision-making in a context where all aspects of care are free at the point of delivery, but where anti-malarial treatment is not available from community health workers based at village health posts. Previous quantitative research in the district identified three key important findings: compared to those living near the hospital, those living in remote villages were less likely to attend a health facility; they were more likely to delay attendance at health facility; and they experienced greater costs associated with attending a health facility [10]. Qualitative analysis adds depth and complexity to these findings. In addition to recognizing the need for seeking formal care outside the home which have been discussed in a another previous paper [13], participants’ accounts of decision-making considerations and processes highlight the interaction of distance to facilities and the anticipated costs related to this, with access to resources to meet costs and decision-making autonomy.

### Distance and costs: interrelated influences on treatment-seeking

Quantitative survey data from the Chikwawa district indicate that those living in remote villages were less likely to attend a formal health facility within 24 h of fever onset compared to those in central villages [10]. The current qualitative data reveals some of the potential reasons for this finding. All participants engaged in cost minimization by spending a period of assessment to judge the severity of the child’s illness before acting, in order to avoid unnecessary trips. Caregivers further aimed to avoid incurring transport costs by walking if they did not consider the illness to be severe. Those in remote villages had the extra complexity of having to consider the feasibility and safety of travelling to the health facility, which effectively created a limited time window for deciding to seek treatment. This limited time window, in combination with cost avoidance, are likely to contribute to the greater delays before treatment for those living in remote villages.

Caregivers explained they would incur transport costs to ensure fast treatment if the illness was considered serious. This explains previous findings that those in remote villages experienced greater direct travel costs during the wet season, when illnesses tend to be more severe, compared to the dry season [10]. Despite extensive flooding during the wet season, journey times did not increase for those living in remote villages. Rather they experienced increased direct costs, reflecting the decision to pay for transport rather than walk in order to minimize the risk of a serious illness progressing during the journey.

It was clear that those in remote villages were willing to make financial sacrifices in order to ensure their child received appropriate treatment. However, their concern about distance and minimizing avoidable travel costs contributed to delayed health facility attendance. Health education messages targeting the importance of prompt treatment are likely to fail while caregivers are forced to engage in treatment-seeking strategies that attempt to prevent potentially catastrophic burdens on the household [25–27]. Interventions to bring care closer to individuals’ homes are essential to improve access to appropriate treatment. In Malawi, this is being attempted through placing CHWs in remote villages. Since this study was performed, the inclusion of RDTs and ACT into CHW services is being implemented by the MoH within hard-to-reach villages. While this is aimed at improving equity of access, utilization and promptness of attendance by reducing barriers, including costs, for the poorest and most at risk members of the population there are concerns about this strategy. RDT and drug allocation and distribution to health facility level has faced challenges over the last few years, further compounded by substantial budget cuts at district level across the health system. While there are no known published figures, there are concerns that community level distribution in this setting may compete with, rather than complement, stock levels at facilities.

### Gender and generation: financial autonomy and decision-making power

A major finding of this study was that in addition to the greater costs and lower geographic accessibility faced by those living in remote villages, women within these communities had much more limited financial autonomy and decision-making authority, which was related to traditions within their ethnic group, as well as lower access to resources, as a result of their more economically marginal location. These multiple layers of disadvantage and marginality intersected to reduce access to formal healthcare for febrile children.

The need for caregivers to seek approval and access household resources from husbands before seeking treatment adds a layer of complexity to the decision-making process, which can further delay care seeking by remote villagers. The decision-making pattern, whereby the mother’s role is to inform others, with males and other senior household members taking responsibility for final decision-making has been reported in other settings and has been seen to contribute to delayed treatment-seeking [1, 3, 4, 28]. In Tanzania, didactic health facility based education messages, which targeted mothers of young children and failed to take account of their lack of decision-making authority, were found to further undermine junior women’s positions and reinforce their perceived low status [29]. Such dominant and teacher-centred delivery of health education messages has also been found during antenatal clinics in Malawi [30].

Female elders have previously been demonstrated to have an important role in treatment-seeking decision-making in Malawi [30–32]. Despite this, health education programmes in Malawi rarely seek to involve grandmothers [31]. Involving the wider community, including men and grandmothers in community-based education programmes in Malawi and elsewhere would ensure that all those involved in the treatment-seeking process are included [29, 31, 33, 34]. The role of older women should also be harnessed in behaviour change strategies [35]; involving grandmothers in health education programmes has been seen to lead to improvements in the education messages they give to mothers, and mothers’ health seeking behaviour [36]. Such interventions should be used to challenge, rather than reinforce, the underlying gender and generational inequalities that shape the treatment-seeking process, through working to promote the position of junior women and improve their power and autonomy in the treatment-seeking processes, particularly in remote villages [1, 7, 25, 29]. The finding that women with greater access to income, either through their role in trading household produce or through their own independent income generation activities, were able to respond more quickly when they recognized the need to seek formal care also coheres with other research in sub-Saharan Africa [8]. This contributes towards the evidence that both women’s independent access to and control over income and financial contributions by fathers are significant in promoting child health [7].

Alongside their other tasks, CHWs in remote villages are responsible for delivering health education messages. Previous studies have demonstrated the benefits of education programmes that strive to engage with communities within their own contexts [7, 36, 37]. Lower and delayed attendance at health facilities by those living in remote villages highlights the importance of adding value to the services provided by CHWs at community level by strengthening the delivery of education messages, particularly where CHW are not equipped to provide malaria treatment. However, community-based facilitators, who are likely to share the socio-cultural background with communities, will require awareness raising, training and support to carry out such sensitive work [38].

## Conclusion

This study has illustrated the complex interplay between geographic access and gendered intra-household dynamics in shaping decisions on whether and when formal treatment is sought for febrile children in Chikwawa District. Geographic marginality and cultural traditions intersect in remote areas both to increase the logistical and financial barriers to accessing care and to reduce caretakers’ autonomy to act quickly once they recognize the need for formal care. These findings underscore the importance of exploring the relationship between social and geographic characteristics of specific contexts to identify the range of intersecting constraints on care-seeking in order to identify appropriate strategies to improve access to effective treatment for children with malaria.

## Abbreviations

ACT: artemisinin-based combination therapy
CHW: community health worker
FGDs: focus group discussions
CIIs: critical incident interviews
CAGs: Community Advisory Groups
RDT: rapid diagnostic test

## References

[1] Tolhurst R, Amekudzi Y, Nyonator F, Theobald S (2008). “He will ask why the child gets sick so often”: the gendered dynamics of intra-household bargaining over healthcare for children with fever in the Volta Region of Ghana. Soc Sci Med.

[2] Tolhurst R, Nyonator F (2006). Looking within the household: gender roles and responses to malaria in Ghana. Trans R Soc Trop Med Hyg.

[3] Molyneux CS, Murira G, Masha J, Snow RW (2002). Intra-household relations and treatment decision-making for childhood illness: a Kenyan case study. J Biosoc Sci.

[4] Williams HA, Jones COH (2004). A critical review of behavioral issues related to malaria control in sub-Saharan Africa: what contributions have social scientists made?. Soc Sci Med.

[5] Kishor S, Presser G (2000). Empowerment of women in Egypt’s and links to the survival and health of their infants. Women’s empowerment and demographic processes moving beyond Cairo.

[6] Fantahun M, Berhane Y, Wall S, Byass P, Hogberg U (2007). Women’s involvement in household decision-making and strengthening social capital-crucial factors for child survival in Ethiopia. Acta Paediatr.

[7] Richards E, Theobald S, George A, Kim JC, Rudert C, Jehan K (2013). Going beyond the surface: gendered intra-household bargaining as a social determinant of child health and nutrition in low and middle income countries. Soc Sci Med.

[8] Colvin CJ, Smith HJ, Swartz A, Ahs JW, de Heer J, Opiyo N (2013). Understanding careseeking for child illness in sub-Saharan Africa: a systematic review and conceptual framework based on qualitative research of household recognition and response to child diarrhoea, pneumonia and malaria. Soc Sci Med.

[9] Springer KW, Hankivsky O, Bates LM (2012). Gender and health: relational, intersectional, and biosocial approaches. Soc Sci Med.

[10] Ewing VL, Lalloo DG, Phiri KS, Roca-Feltrer A, Mangham LJ, SanJoaquin MA (2011). Seasonal and geographic differences in treatment-seeking and household cost of febrile illness among children in Malawi. Malar J.

[11] Chuma J, Okungu V, Molyneux C (2010). Barriers to prompt and effective malaria treatment among the poorest population in Kenya. Malar J.

[12] McCombie SC (1996). Treatment seeking for malaria: a review of recent research. Soc Sci Med.

[13] Ewing VL, Tolhurst R, Kapinda A, SanJoaquin M, Terlouw DJ, Richards E (2015). Understanding interpretations of and responses to childhood fever in the Chikhwawa district of Malawi. PLoS ONE.

[14] Mudeka I. “We faced Mabvuto”: a gendered socio-economic history of Malawian women’s migration and survival in Harare (Ph.D thesis), 1940 to 1980. Minneapolis: University of Minnesota; 2011.

[15] Mandala EC (1978). The nature and substance of mang’anja and kololo oral traditions: a preliminary survey. The Society of Malawi Journal..

[16] Phiri KM (1983). Some changes in the matrilineal family system among the Chewa of Malawi since the nineteenth century. J Afr Hist.

[17] Kitzinger J (1994). The methodology of Focus Groups: the importance of interaction between research participants. Sociol Health Illn.

[18] Khan ME, Manderson L (1992). Focus groups in tropical diseases research. Health Policy Plan.

[19] Patton MQ (1990). Qualitative evaluation and research methods.

[20] Mays N, Pope C (1995). Qualitative research: rigour and qualitative research. BMJ.

[21] Roca-Feltrer A, Lalloo DG, Phiri K, Terlouw DJ (2012). Rolling malaria indicator surveys (rMIS): a potential district-level malaria monitoring and evaluation (M&E) tool for program managers. Am J Trop Med Hyg.

[22] Sandelowski M (1995). Sample size in qualitative research. Res Nurs Health.

[23] Britten N (1995). Qualitative research: qualitative interviews in medical research. BMJ.

[24] Richie J, Spencer L, O’Connor W. Qualitative research practice: a guide for social science students and researchers. In: Richie J, Lewis J, editors. London: SAGE Publications Ltd; 2003.

[25] Ribera JM, Hausmann-Muela S (2011). The straw that breaks the camel’s back. Redirecting health-seeking behavior studies on malaria and vulnerability. Med Anthropol Q.

[26] Chuma J, Gilson L, Molyneux C (2007). Treatment-seeking behaviour, cost burdens and coping strategies among rural and urban households in Coastal Kenya: an equity analysis. Trop Med Int Health.

[27] Chuma JM, Thiede M, Molyneux CS, Chuma JM, Thiede M, Molyneux CS (2006). Rethinking the economic costs of malaria at the household level: evidence from applying a new analytical framework in rural Kenya. Malar J.

[28] Orubuloye IO, Caldwell JC, Caldwell P, Bledsoe CH (1991). The impact of family and budget structure on health treatment in Nigeria. Health Transit Rev.

[29] Montgomery CM, Mwengee W, Kong’ong’o M, Pool R (2006). ‘To help them is to educate them’: power and pedagogy in the prevention and treatment of malaria in Tanzania. Trop Med Int Health.

[30] Launiala A. Prevention of malaria in pregnancy in Malawi (PhD Thesis). University of Tampere, Faculty of Medicine; 2010.

[31] Bezner Kerr R, Dakishoni L, Shumba L, Msachi R, Chirwa M (2008). ”We grandmothers know plenty”: breastfeeding, complementary feeding and the multifaceted role of grandmothers in Malawi. Soc Sci Med.

[32] Tolhurst R, Theobald S, Kayira E, Ntonya C, Kafulafula G, Nielson J (2008). ‘I don’t want all my babies to go to the grave’: perceptions of preterm birth in Southern Malawi. Midwifery.

[33] Gender influences on child survival, health and nutrition: a narrative review. http://www.unicef.org/nutrition/files/Gender_Influences_on_Child_Survival_a_Narrative_review.pdf.

[34] Franckel A, Lalou R, Franckel A, Lalou R (2009). Health-seeking behaviour for childhood malaria: household dynamics in rural Senegal. J Biosoc Sci.

[35] Aubel J (2012). The role and influence of grandmothers on child nutrition: culturally designated advisors and caregivers. Matern Child Nutr.

[36] Aubel J, Toure I, Diagne M (2004). Senegalese grandmothers promote improved maternal and child nutrition practices: the guardians of tradition are not averse to change. Soc Sci Med.

[37] Rath S, Nair N, Tripathy PK, Barnett S, Rath S, Mahapatra R (2010). Explaining the impact of a women’s group led community mobilisation intervention on maternal and newborn health outcomes: the Ekjut trial process evaluation. BMC Int Health Hum Rights.

[38] Theobald S, MacPherson E, McCollum R, Tolhurst R (2015). Close to community health providers post 2015: realising their role in responsive health systems and addressing gendered social determinants of health. BMC Proc.

